# The impact of medication side effects on adherence and persistence to hormone therapy in breast cancer survivors: A quantitative systematic review

**DOI:** 10.1016/j.breast.2022.04.010

**Published:** 2022-05-14

**Authors:** Leanne Fleming, Sommer Agnew, Nicola Peddie, Megan Crawford, Diane Dixon, Iain MacPherson

**Affiliations:** a University of Strathclyde, George Street, Glasgow, UK; b University of Aberdeen, Kings College, Aberdeen, UK; c University of Glasgow, University Avenue, Glasgow, UK

**Keywords:** Adjuvant hormone therapy, Breast cancer, Side effects, Endocrine therapy, Tamoxifen, Aromatase inhibitor, Adherence, Persistence, Quantitative

## Abstract

**Background:**

Hormone Therapy (HT) is recommended for most women with HR-positive primary breast cancer. When taken as intended, HT reduces breast cancer recurrence by 40% and mortality by one-third. The recommended duration of treatment ranges from 5 to 10 years depending on risk of recurrence and the specific HT regimen. However, recent data indicates that rates of HT non-adherence are high and research suggests this may be due to the impact of HT side effects. The contribution of side effects to non-adherence and non-persistence behaviours has rarely been systematically explored, thereby hindering the implementation of targeted intervention strategies. Our aim is to identify, evaluate and summarise the relationship between HT side effects and patterns of adherence and persistence.

**Methods:**

Electronic searches were conducted from inception and were completed by September 2021, utilising Cochrane CENTRAL, Medline, Embase, Web of Science and PsycINFO databases. Searches included a combination of terms related to breast cancer, adherence, hormone therapy and side effects.

**Results:**

Sixty-two eligible papers were identified and study quality varied by study type. Most observational and cross-sectional studies were rated good quality, whereas most controlled intervention studies were rated fair quality. Three studies were rated poor quality. The most frequently measured side effects were pain, low mood, hot flashes, insomnia, anxiety, fatigue, weight gain, concentration/memory problems.

**Conclusions:**

This review identified a lack of consistency in the measurement of adherence and the definition of persistence across studies. The instruments used to measure side effects also varied significantly. This variation and lack of consistency makes it difficult to evaluate and summarise the role of HT side effects in HT adherence and persistence behaviour.

## Highlights

• Hormone Therapy (HT) reduces breast cancer recurrence and mortality yet HT non-adherence and non-persistence is high.
• The experience of HT side effects may contribute to HT non-adherence and non-persistence.
• Pain, low mood, hot flashes, insomnia and poor concentration are amongst the most commonly reported HT side-effects.
• Assessment tools measuring HT side effects vary significantly across studies, making cross-study comparisons difficult.
• There is a lack of cross-study consistency in defining persistence and measuring adherence.
• Poor cross-study consistency limits understanding of the role of HT side effects in adherence and persistence behaviour.

## 1 Introduction

Approximately 85% of all breast cancers are hormone-receptor-positive [1] and as such, are treatable with Hormone Therapy (HT). HT works by interfering with signalling through the estrogen receptor, either by binding to the receptor directly, as is the case with Selective Estrogen Receptor Modulators (SERMS) (e.g. Tamoxifen) or by reducing production of estrogen, as is the case with Aromatase Inhibitors (AI) (e.g. Letrozole, Anastrozole and Exemestane). HT is recommended for almost all women with HR-positive primary breast cancer as, when taken as intended, HT reduces breast cancer recurrence by 40% and mortality by one-third [2,3]. HT is typically started close to the time of diagnosis unless adjuvant chemotherapy is also planned, in which case HT begins after completion of chemotherapy. The recommended duration of treatment can range from 5 to 10 years depending on the risk of recurrence and the specific HT regimen. However, recent data indicates that rates of HT non-adherence are high, with approximately half of all women prescribed HT taking less than 80% of their prescribed dose [4,5]. Further, up to 50% discontinue their HT regimen by the fifth year of prescription [6]. Given the importance of adherence to and persistence with HT for minimising cancer recurrence, understanding the mechanisms that impact adherence and persistence behaviour is a key priority to promote cancer-free survival.

In HT literature, a distinction is frequently made between initiation adherence (how many individuals start treatment), medication adherence (once started, how many take the medication as prescribed in terms of dose, timing and frequency), and medication persistence (the duration of treatment from initiation to discontinuation) [7]. Non-adherence can be categorised as intentional (an individual deciding not to take their medication as prescribed), and unintentional (an individual forgetting to take their medication or misunderstanding the instructions) [4]. Previous research on HT adherence and persistence has largely focused on the predictive value of sociodemographic, clinical and psychosocial factors [5,8]. Poor HT adherence has been associated with older age [9], greater number of other medications prescribed for comorbidities [10], switching between HT prescriptions [11], and HT side effects [8,12]. Although sociodemographic and clinical variables (age, number of other prescriptions, prescription alterations) seem to negatively impact HT adherence and persistence [11,13,14], the only consistent predictors to emerge from this previous work are HT side effects [12,15,16]. It is the identification of these HT side effects that offer the most promise as intervention targets for adherence and persistence behaviour change.

Sleep disturbance, fatigue, joint pain and menopausal symptoms are amongst the most frequently reported side effects of HT to affect adherence and persistence behaviour [5,8,17,18]. This may be because the daily experience of these adverse effects outweighs the potential benefits of HT for some patients [9]. However, previous studies on the role of side effects in adherence to HT, report only the presence or absence of an overall side effect profile. No study has systematically explored how individual side effects may influence HT adherence and persistence, or which specific side effects may have the most profound impact [[19], [20], [21]]. This is problematic, because unlike sociodemographic and clinical factors, HT side effects may be amenable to behaviour change interventions. Lack of clarity over the contribution of specific side effects to HT non-adherence and non-persistence prevents delivery of appropriate, targeted intervention strategies.

Two systematic reviews [8,22] and one scoping review [23] exploring HT use in breast cancer, have been published in the last five years. Neither of these reviews were designed to specifically explore the impact of individual HT side effects on adherence and persistence behaviour. Moon et al. (2017) [8] and Zhu et al. (2019) [23] did report prevalence data of the most common side effects experienced by women taking HT but did not conduct a detailed synthesis of the relationship between these and HT adherence and persistence. Similarly, Lambert [22] explored a broad range of personal, social, and structural patient related factors that influence adherence, rather than specifically focusing on side effects. Therefore, the aim of this review is to identify, evaluate and summarise the relationship between HT side effects and patterns of adherence and persistence. This will facilitate the identification of key intervention targets to promote HT adherence and persistence and as such, has potential to improve cancer outcomes.

## 2 Method

The protocol for this systematic review was registered on the PROSPERO database on August 13, 2020 (CRD42020192481) https://www.crd.york.ac.uk/prospero/display_record.php?ID=CRD42020192481. Reporting has been conducted as per the PRISMA statement [24].

### 2.1 Information sources and search

Electronic searches were completed by September 3, 2021, using the following databases from inception: Cochrane CENTRAL, Medline, Embase, Web of Science and PsycINFO. We contacted authors of conference abstracts to request full-text papers and searched grey literature databases and trial registries for unpublished research. The search strategy was adapted from Moon et al. (2017) [8]. A combination of search terms related to 1) breast cancer, 2) adherence 3) HT and 4) side effects were included. Terms related to ‘symptoms’ and ‘toxicities’ were not included. On reflection, whilst these may have been a useful addition, we are confident that their exclusion has not made a significant difference to the results as these terms are most often used to discuss an overall side effect profile rather than specific side effects. A full copy of the search strategy has been included as a supplementary file. As outlined in the protocol, our intention was to undertake a mixed methods review of the literature on the impact of HT side effects on adherence and persistence. However, the initial search generated such a significant volume of quantitative and qualitative studies that we decided it was more comprehensive to undertake two separate reviews. The qualitative review has been published separately [25].

### 2.2 Eligibility criteria

The inclusion/exclusion criteria were adapted from the criteria used by Moon et al. (2017) [8] but amended to focus specifically on the impact of side effects on HT adherence and persistence. Studies were included if they: (i) recruited female participants aged 18 years or older who were prescribed HT for primary breast cancer; (ii) were trials or were conducted in clinical practice, and (iii) presented statistical tests of association between HT adherence or persistence and side effects as a correlate or predictor. Studies were excluded if they only included patients with ductal carcinoma in situ (DCIS) or Stage IV cancer. Studies using an intervention to improve adherence were only assessed if they reported side effects, but only data from the control group was extracted. Studies were also excluded if they were not available in English, a full text version was unavailable, did not include primary data, were related to screening or diagnosis, or used non-human subjects.

### 2.3 Study selection

All screening was conducting using the Covidence platform (Melbourne, Australia), an online tool used to manage systematic review screening and data extraction. All references were uploaded to this platform, and after the removal of duplicates, all remaining titles generated from the search were screened. Next, titles and abstracts were screened using the inclusion/exclusion criteria, and if an abstract did not provide sufficient exclusion information, the article was obtained for full text screening. If a full text paper was unavailable, authors were contacted to request a copy. All screening was performed independently by two reviewers (NP and SA) and disagreements were resolved through discussion or by a third reviewer (LF), when consensus could not be reached. A Cohen's kappa statistical test was calculated to determine the level of agreement between reviewers. The level of agreement between the authors was substantial (K = 0.61).

### 2.4 Data extraction

Data were extracted from the method and results sections of the included studies using a data extraction form. The following information was extracted: author, year, study design, drug treatment, country, participant characteristics (sample size, age, cancer stage, reported demographics), reported side effects, adherence measures, and effect of side effects on adherence and/or persistence. Data extraction was conducted independently by NP and SA.

### 2.5 Risk of bias

Quality assessment of the included papers was conducted using the National Heart, Lung and Blood Institute (NHLBI) quality assessment tool. The NHLBI quality assessment tool offers a method of critically appraising the quality and risk of bias in relevant studies. Studies are evaluated holistically, considering the risk of bias introduced by potential flaws. For this reason, a numerical score is not produced by this tool. An additional item was added to the NHLBI quality assessment tool to assess if adherence/persistence had been clearly defined and appropriately measured. Studies were categorised as either good, fair, or poor quality, based on independent reviewer judgement (NP and SA). When ratings differed between reviewers, discussion took place to reach agreement. When consensus could not be reached, a third reviewer (LF) was consulted.

### 2.6 Data analysis

A Harvest Plot [26] was constructed to assist the process of synthesis and provide a visual representation of evidence of the relationship between the reported side effect(s) and HT adherence and persistence. Heterogeneity in the measurement of side effects and adherence behaviour and lack of clarity regarding type of HT drug [27,28], precluded effective meta-analyses, therefore a narrative synthesis is presented.

## 3 Results

### 3.1 Study selection

**Fig. 1:**
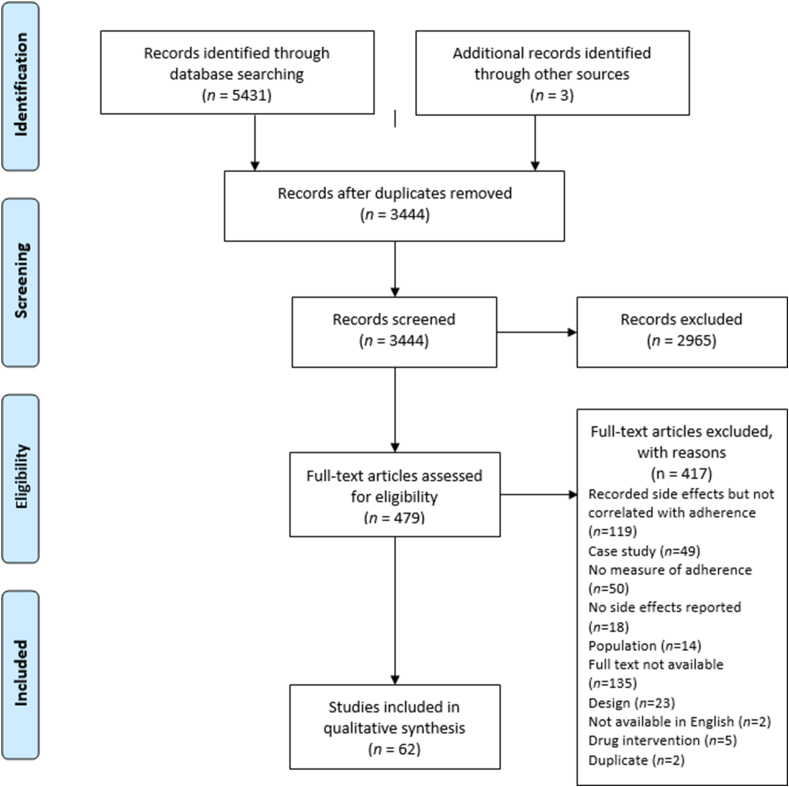
Prisma flow diagram.

In total, 5341 records were identified, and 3444 papers remained after removal of duplicates. Screening of titles and abstracts identified 479 papers for full text review. Of these full text papers, 62 met the inclusion criteria and there was complete agreement amongst the reviewers about the included papers. The reasons for exclusion are shown in the PRISMA flow diagram (Fig. 1).

### 3.2 Study characteristics

Study characteristics are presented in Table 1. Sample sizes ranged from 25 to 32,311. The 62 included papers reflected 59 unique datasets. Most studies (N = 35) included prescriptions of both drug categories (AI and SERM) but did not distinguish between these when reporting side effect data. Sixteen studies focused on AI prescriptions, 9 focused on SERM prescriptions, and 2 studies did not specify HT type. Study designs included cohort (N = 29), cross-sectional (N = 15), observational (N = 9), randomized controlled trial (N = 8) and repeated measures (N = 1). Studies measured either adherence (N = 32), persistence (N = 21), or both (N = 9).

**Table 1:** Study characteristics.

Study reference	Country	Setting	Drug category (SERM or AI)	Specific drug(s)	Sample Size Recruited (Sample Size Analysed)	Sample demographics	Cancer Type/Stage	Adherence and/or Persistence	Adherence/persistence measure	Side effects measure
Bedi et al. (2020) [29]	USA	Clinical practice	Both	Tamoxifen, Anastrozole/Exemestane/Letrozole	1399 (1339)	Age: Median = 49, (Range = 21–64) Ethnicity: 53% White, 44% African American	Hormone receptor-positive breast cancer Stage 0: 12%, Stage 1: 41%, Stage 2: 2%, Stage 3: 38%, Stage 4: 6%, unknown: 1%	Persistence	HT usage duration	Use of medications that treated known side effects (medical records)
Bender et al. (2014) [21]	USA	Cancer research centre	Both	Tamoxifen and Anastrozole, Letrozole, and Exemestane	91 [91]	Age: *M* = 56.7 (*SD* = 9.7) Ethnicity: Caucasian (*N* = 88), Other: (*N* = 1)	Hormone receptor-positive breast cancer Stage 1: *N* = 54 Stage 2/3: *N* = 37	Adherence	AARDEX microelectronic monitoring system (MEMS™) cap.	Various cognitive function tests Beck Depression Inventory (BDI)-II Profile of Mood States (POMS) Breast Cancer Prevention Trial (BCPT) Symptom Checklist-Physical Functioning subscale
Bowles et al. (2012) [30]	USA	Clinical practice	Both	Tamoxifen and Anastrozole, Letrozole, and Exemestane	693 (538)	Age: Adherer *M* = 64, (*SD* = 8.4) Discontinuer *M* = 65 (*SD* = 9.7) Ethnicity: White (*N* = 483), Other (*N* = 52)	HER receptor (positive: *N* = 165, negative: *N* = 373) Stage I (*N* = 304), Stage IIA: (*N* = 164), Stage IIB (*N* = 70)	Persistence	Self-reported still using HT, usage of >5 years	Self-reported yes/no
Brett et al. (2018) [31]	UK	Hospital	Both	Tamoxifen and Anastrozole, Letrozole, and Exemestane	292 (211)	Age: Median = 63, (Range = 36–85) 98% White British, 2% Other	In breast only 146 (69%) In breast and lymph nodes 65 (31%)	Adherence	Medical Adherence Report Scale (MARS-5)	Self-report presence of side effects
Brier et al. (2015) [88]	USA	Breast cancer clinic	AI	Letrozole, Anastrazole, Exemestane	235 (235)	Age: Range = 35-84 Ethnicity: 85.1% White, 14.9% Non-white	Hormone receptor positive breast cancer	Adherence	Yes/no self-report, Modified Morisky Medication Adherence Scale-8 (MMAS-8), Visual Analog Scale (VAS) indicating % of use over past month	Hospital Anxiety and Depression Scale (HADS), Measures of pain, AI side-effects, emotional wellbeing, sleep and fatigue, physical functioning and physical activity
Brier et al. (2018) [32]	USA	Breast cancer clinic	AI	Anastrazole, Letrozole, Exemestane	862 (509)	Age: <55 (*N* = 109), 55–70 (*N* = 299), >70 (*N* = 105) Ethnicity; 80.4% White, 19.7% Non-white	Stage 1: 56.8% Stage 2: 28.5% Stage 3: 13%	Adherence	Non-adherence defined as a treatment interruption and/or premature discontinuation	Penn Arthralgia Aging Scale (PAAS),
Brier et al. (2017) [33]	USA	Breast cancer clinic	AI	Anastrozole, Letrozole, or Exemestane	437 (437)	Age: >65 (*N* = 138), 55–65 (*N* = 201), <55 (*N* = 98) Ethnicity: 82.6% White, 17.4% Non-White	Stage I-III hormone receptor positive breast cancer	Adherence	Non-adherence defined as a treatment interruption and/or premature discontinuation	Health Beliefs and Medication Adherence in Breast Cancer (HBMABC) scale, Brief Pain Inventory (BPI
Brier et al. (2018)b [34]	USA	Teaching hospital	AI	Anastrozole, Letrozole, or Exemestane	862 (506)	Age: Range = 56-70 Ethnicity: 80.4% White, 19.7% Non-White	Stage I-III hormone receptor positive breast cancer	Adherence	Non-adherence defined as a treatment interruption and/or premature discontinuation	HBMABC, BPI, HADS
Bright et al. (2016) [35]	USA	Community	Both	Tamoxifen, Anastrazole, Exemestane, or Letrozole	2086 (1371)	Age: *M* = 56 Ethnicity: 93% Non-Hispanic white	Early-stage breast cancer	Adherence	Adapted MMAS-8	BCPT Symptom Scales with added items for AI side effects, added items for barriers to taking HT
Chim et al. (2013) [36]	USA	Hospital	AI	Anastrozole, Letrozole, Exemestane	501 (437)	Age: 31.6% > 65, 46.0% 55–65, 22.4% < 55 Ethnicity: 82.4% White, 17.6 Non-white	Stage I: 38.6% Stage II: 49.0% Stage III: 12.4%	Adherence	Premature discontinuation	BPI
Cluze et al. (2012) [37]	France	Community	SERM	Tamoxifen	218 (196)	Age: *M* = 37 (*SD* = 3.5)	Hormone receptor-positive breast cancer Stage 1: 33% Stage 2: 51% Stage 3: 15% Grade 1: 14% Grade 2: 53% Grade 3: 32%	Persistence	Tamoxifen interruption-2+ months without dispensed prescription	Centre for Epidemiologic Studies–Depression (CES-D), self-reported Tamoxifen symptoms
Corter et al. (2018) [38]	New Zealand	Oncology clinic	Both	Tamoxifen and Anastrozole, Aetrozole, and Exemestane	125 (120)	Age: *M* = 56 years, (*SD* = 10.5; Range = 31–88 years) Ethnicity: 65% NZ European, 14% Māori/Pacific Islander, 21% “Other”	HER receptor: Positive 15%; Negative 85% Grade I 15%; Grade II 56%; Grade III 29%	Adherence	Self-reported no. Of missed doses in past 30 days (“nonadherent” if they reported missing any dose of their ET during the last 30 days)	BCPT Symptom Scales
Cuzick et al. (2007) [39]	UK	Hospital	Both	Anastrozole and Tamoxifen	6000 (6000)	Not reported	84% hormone receptor positive 61% node negative	Persistence		
Demissie et al. (2001) [40]	USA	Hospital	SERM	Tamoxifen	388 (303)	Age: *M* = 67.7 (*SD* = 8.7)	76% estrogen receptor– positive 63% had stage I breast cancer	Persistence	Self-reported Tamoxifen use: defined as taking tamoxifen at any time during the study period	2 side-effect variables with yes/no responses: hot flashes alone and any side effects
Font et al. (2019) [41]	Spain	Clinical practice	SERM	Tamoxifen	2413 (2413)	Age: <50 (*N* = 676), 50–69 (*N* = 1122), >69 (*N* = 615)	Positive hormone receptors (ER+) breast cancer Stage I 965; Stage II 1011; Stage III 437	Adherence	Proportion of days covered by a filled drug prescription over the treatment period (5 years), 80% considered satisfactory adherence	Self-reported Adverse effects-yes/no
Gao et al. (2018) [42]	China	Hospital	Both	Tamoxifen, Anastrozole or Letrozole	1110 (699)	Age: Adherent: 16.3% < 40 (*N* = 72), 68.9% 40–59 (*N* = 304), 14.7% ≥ 60 (*N* = 65) Non-adherent: 13.2% < 40 (*N* = 34), 68.6% 40–59 (*N* = 177), 18.2% ≥ 60 (*N* = 47) Ethnicity: Adherent 94.8% Han (*N* = 418), 58.7% Minorities (*N* = 259) Non-adherent: 95.3% Han (*N* = 246), 4.7% Minority (*N* = 12)	ER- and/or PR-positive status	Both	Compliance-adherence to prescribed medications and interruption of >180 days; persistence- continuation of endocrine therapy for at least 5 years.	Self-reported Adverse effects-yes/no
Grossman et al. (2016) [43]	USA	Community	Both	85% Aromatase Inhibitors (AIs) and 15% Tamoxifen	40 [40]	Age: *M* = 59.3 (*SD* = 6.67) Ethnicity: 92.5% Caucasian (*N* = 37) 0% African American, 0% Hispanic, 2.5% Asian/Pacific Islander (*N* = 1), 2.5% American Indian (*N* = 1), 2.5% Mixed (*N* = 1)	Stage 0 3 (7.7%) Stage 1 27 (69.2%) Stage 2 7 (17.9%) Stage 3 2 (5.1%)	Adherence	MEMS cap: 80% adherence cut-off, 2 self-report items	CES-D Psychological Symptom Distress Scale
Hadji et al. (2014) [44]	Germany	Clinical setting	AI	Anastrazole	2210 (1916)	Age: *M* = 65 (*SD* = 8)	HR + early breast cancer	Adherence	Defined as compliant when both doctor and patient rated compliance to be ≥ 80%	Rheumatoid Arthritis Symptom Questionnaire (RASQ)
Helland et al. (2019) [45]	Norway	University hospital	SERM	Tamoxifen	220 (220)	Age: *M* = 48.69 (median = 48.08, Range = 24–84)	Grade: G1: 30 (13.60%) G2: 113 (51.40%) G3: 64 (29.10%) Unknown: 13 (5.90%) Receptor status: HER2+ 37 (16.80%) HER2− 182 (82.70%) Not reported 1 (0.50%)	Both	Medication possession ratio (MPR) (80% cutoff)	Functional Assessment of Cancer Therapy-Endocrine Symptoms (FACT-ES)
Henry et al. (2012) [46]	USA	Cancer Centre	AI	Exemestane and Letrozole	503 (500)	Age: Median = 59 (Range = 35–89) Ethnicity: 88.2% White (*N* = 441); 9.2% Black (*N* = 46); 2.6% Other (*N* = 13)	Stage 0 to III HR-positive breast cancer	Persistence	Duration of usage	Modified Health Assessment Questionnaire (HAQ) and pain visual analog scale (VAS)
Henry et al. (2010) [47]	USA	Cancer Centre	AI	Exemestane and Letrozole	29 [25]	Age: Median = 61 (Range = 47–83) Ethnicity: 86% Non-Hispanic White (*N* = 25), 7% Hispanic White (N = 2), 3% Black (*N* = 1), 3% Asian: (*N* = 1)	Early stage hormone receptor positive breast cancer	Persistence	Early discontinuation	HAQ, VAS
Henry et al. (2013) [48]	USA	Cancer Centre	AI	Exemestane and Letrozole	503 (432)	Age: Median = 59 (Range = 35–89) Ethnicity: 88.4% White (*N* = 413), 9% Black (*N* = 42), 2.6% Other (*N* = 12)	HR-positive stage 0–III breast cancer	Persistence	Early treatment discontinuation	Dropout due to adverse effects
Henry et al. (2017) [49]	USA	University Hospital	Both	Tamoxifen or a third-generation AI (Anastozole, Exemestane, Letrozole)	115 (115)	Age: Median = 62 [[41], [42], [43], [44], [45], [46], [47], [48], [49], [50], [51], [52], [53], [54], [55], [56], [57], [58], [59], [60], [61], [62], [63], [64], [65], [66], [67], [68], [69], [70], [71], [72], [73], [74], [75], [76], [77], [78], [79]]	Stage 0-III breast cancer	Adherence	MARS-5	Self-report pain Likert scale & questionnaire
Hershmann et al. (2016) [50]	USA	Medical centre, teaching hospital	Both	Not specified	601 (523)	Age: 50.5% < 60 (*N* = 264), 49.5% > 60 (*N* = 259) Ethnicity: 75.3% White (*N* = 394), 7.3% Black (*N* = 38), 17.4% Other (*N* = 91)	Stage I: 317 (60.6%) Stage II/III: 206 (39.4%) HER receptor: Negative: 465 (89.9%) Positive: 52 (10.1%)	Persistence	Gap between filling HT prescriptions (≥90 day gap considered non-persistent)	Functional Assessment of Cancer Therapy-Breast (FACT-B), Treatment Satisfaction Questionnaire for Medication (TSQM)
Hsieh et al. (2015) [51]	Taiwan	Clinical practice	Both	Not specified	32,311 (32,311)	*M* = 52.3, ±11.6	Newly diagnosed Breast Cancer	Persistence	MPR (gap of 180 days between prescriptions considered interrupted)	Use of medications that treated known side effects (medical records)
Iacorossi et al. (2016) [52]	Italy	National cancer institute	Both	Not specified	151 (151)	Age: 48.3% < 53(*N* = 73) 49.7% > 53(*N* = 75), 92.1% < 70(*N* = 139), 6.0% > 70 years (N = 9)	Outpatients diagnosed with breast cancer	Adherence	MMAS-8	Distress Thermometer
Jackisch et al. (2019) [53]	Germany	Breast cancer clinic	AI	Anastrazole	4923 (4844)	Age: 49% ≤ 65 (*N* = 1035), 51% > 65 (*N* = 1079)	Early breast cancer ER+ 4298 (98.9%) Grade: G1: 602 (13.7%) G2: 2924 (66.7%) G3: 820 (18.7%)	Both	Self-reported daily intake- 80–100% considered compliant	European Organization for Research and Treatment of Cancer (EORTC) symptom scale
Kadakia et al. (2016) [54]	USA	Teaching hospital	AI	Exemestane and Letrozole	503 (500)	*M* = 59 88% White, 12% Black/other	Stage 0–III hormone receptor-positive breast cancer	Persistence	Early discontinuation	EuroQOL VAS, CES-D, HADS, BCPT Symptom checklist
Kahn et al. (2007) [55]	USA	Clinical practice	SERM	Tamoxifen	881 (881)	Age: 26% < 50 (*N* = 228), 44% 50–65 (*N* = 386), 30% 65+ (*N* = 267) Ethnicity: 5% Hispanic white/other (*N* = 40), 85% Non-Hispanic white (*N* = 747), 4% Non-Hispanic other (*N* = 35), 7% Black (*N* = 59)	Stage I: 54% Stage II: 40%	Persistence	Persistent-continued HT for at least 4 years	Patient questionnaire
Kidwell et al. (2014) [56]	USA	University hospital	AI	Exemestane and Letrozole	500 (449)	Age: *M* = 59.0 Ethnicity: 89.3% White (*N* = 401), 10.7% Black/Other (*N* = 48)	Stage I: 234, 52.3% Stage II: 143, 32.0%. Stage III: 43, 9.6%	Persistence	Persistent group-continued HT for 1+ years	CES-D, HADS, Pittsburgh Sleep Quality Index (PSQI), BCPT Symptom checklist
Kimmick et al. (2015) [57]	USA	Breast cancer clinic	Both	Not specified	143 (112)	Age: *M* = 64 (*SD* = 9) Ethnicity: 81.3% White (*N* = 91), 15.2% African American (*N* = 17), 3.6% Other (*N* = 4)	Stage I: 43 (38.4) Stage II: 56 (50.0) Stage III: 13 (11.6)	Adherence	MMAS-8 and 8 additional items	Brief Fatigue Inventory (BFI), BPI, Menopause Specific Quality of Life Questionnaire
Kool et al. (2014) [58]	Netherlands	Hospital	AI	Letrozole	471 (339)	Age: *M* = 59.81 (*SD* = 9.36)	Stage: T1 149 (46%) T2 154 (47.5%) T3 17 (5.2%) T4a-c 2 (0.6%) T4d 1 (0.3%)	Adherence	Self-report, compliant If they reported never forgetting to take medication	EORTC Quality of Life (QLQ) & Breast Cancer Specific Module (BR23)
Kostev et al. (2013) [59]	Germany	Clinical practice	SERM	Tamoxifen	7792 (3620)	Age: Switch: *M* = 60.0 (*SD* = 14.1) No switch: *M* = 60.6 (*SD* = 13.2)	Diagnosed with breast cancer	Persistence	Discontinuation-90 days not covered by prescription record	Medical records & diagnosis of osteoporosis, depression
Kyvernitakis et al. (2014) [20]	Germany	Hospital	Both	Not specified	180 (180)	Age: *M* = 63.2 (*SD* = 8.8)	Stage: T1 107 61.5% T2 54 31.0% T3 10 5.7% T4 3 1.7%	Adherence	Self-report, prescription records & medical records: 80% tablet intake considered adherent	Menopause Rating Scale (MRS)
Lash et al. (2006) [19]	USA	Hospital	SERM	Tamoxifen	586 (462)	Age at diagnosis: 58% 70–79, 25% 65–69	Estrogen-receptor positive or indeterminate breast cancer	Persistence	Discontinued within 5 years	Mental Health Index (MHI-5), Physical Function Index (PF-10)
Li et al. (2019) [60]	China	Hospital	Both	Tamoxifen, Anastrozole plus Goserelin therapy	62 [62]	Age: Median = 41 (Range = 9–51)	Tumor grade: Intermediate: ADD 20 (60.6%) TAM 18 (62.1%) High ADD 3 (9.1%) TAM 2 (6.9%) Unknown ADD 10 (30.3%) TAM 9 (31.0%) HER-2 status, n (%): Negative: ADD: 26 (78.8%) TAM: 24 (82.8%) Positive ADD: 4 (12.1%) TAM: 3 (10.3%) Unknown ADD: 3 (9.1%) TAM: 2 (6.9%)	Persistence	Withdrawal due to adverse effects	FACT-B, Brief Index of Sexual Functioning for Women (BISF–W)
Liu et al. (2013) [61]	USA	Clinical practice	Both	Tamoxifen and AI (not specified)	921 (303)	Age: *M* = 51.2(*SD* = 9.4) Ethnicity: 34% White (*N* = 103), 49.2% Less-acculturated Latina (*N* = 149), 5% More-acculturated Latina (*N* = 15), 2% African-American (*N* = 6), 8.3% Asian/Pacific Islander: (*N* = 25), 1.6% Other (*N* = 5)	Stage I: 90 (29.7%)Stage II/III: 213 (70.3%)	Adherence	Self-reported HT use 36 months post-diagnosis	Side effect data from medical records
Llarena et al. (2015) [62]	USA	Hospital	SERM	Tamoxifen	703 (515)	Age: Median = 41 Ethnicity: 70.5% White (*N* = 363), 12.2% Black (*N* = 63), 7.6% Hispanic (*N* = 39), 7.4% Asian (*N* = 38), 1.9% Other (*N* = 10),0.4% Missing (*N* = 2)	Stage 0-III, estrogen receptor–positive and/or progesterone receptor–positive breast cancer Stage 0: 99 (19.2%) Stage I: 156 (30.3%)Stage II: 183 (35.5%) Stage III:77 (15.0%)	Persistence	Early discontinuation	Medical records
Mao et al. (2020) [63]	USA	Clinical practice	Both	SERMs And AIs	363 (201)	Age: *M* = 59.2 (*SD* = 11.4) 72.1% White (*N* = 145), 15.9% Asian (*N* = 32), 8.5% Black/African American (*N* = 17), 2% Hispanic/Latino (*N* = 4), 1.5% All others (*N* = 3)	Stage I 128 (63.7%) Stage II 55 (27.4%) Stage III 18 (9.0%)	Persistence	Treatment interruption/discontinuation	Provider notes reviewed for side effects, symptom severity categorised as none–minimal, mild–moderate, or severe
Markovitz et al. (2017) [64]	USA	Radiation clinic	Both	Not specified	203 (133)	*M* = 68.4 years (*SD* = 12.74) 93.2% White (N = 124), 3.8% African‐American, 3.8% Asian‐American, American‐Indian, 3.8% Other, 3% missing	Stage 0: 0.8%, Stage I: 40.6%, Stage II: 25.6%, Stage III: 11.3%, Stage IV: 3.8%, 11.3% reported not knowing the stage	Adherence	4-item MMAS	CES-D, POMS, self-report survey of physical symptoms
Moon et al. (2019) (4)	UK	Clinical practice	SERM	Tamoxifen	345 (345)	Age: *M* = 51.7 (*SD* = 10.3, Range = 30–90) 95% Ethnicity: White (*N* = 325), 5% Other (*N* = 19)	Stage I: 138 (41%) Stage II: 153 (45%) Stage III: 39 (11%) Unsure 11 (3%)	Adherence	MARS- ≤ 24 considered nonadherent	HADS, FACT-ES additional concerns subscale
Nabieva et al. (2018) [65]	Germany	Breast cancer centre	AI	Letrozole	5045 (3887)	Age: Persistent: *M* = 64.7 (*SD* = 8.3) Non-persistent: *M* = 65.8 (*SD* = 8.7)	Hormone receptor positive breast cancer	Adherence	Self and clinician-report	Treatment ending due to side effects
Nestoriuc et al. (2016) [66]	Germany	Breast care centre	Both	Not specified	191 (111)	Age: *M* = 55.5 (SD = 11.0, Range = 26–79)	Stage 0: 3 (2.7%) Stage I: 58 (52.3%) Stage II: 34 (30.6%) Stage III: 14 (12.6%) Stage IV: 2 (1.8%)	Both	Self-report questionnaire	Modified General Assessment of Side-effects Scale (GASE), HADS
Pan et al. (2018) [15]	Germany	Breast care centre	Both	Not specified	116 (116)	Age: *M* = 55.4 (*SD* = 9.97, Range = 26–79)	HER receptor positive Stage: Stage 0 3.4%; Stage 1: 51.7%; Stage 2: 31%; Stage 3: 10.3%; Stage 4: 3.4%	Adherence	Validated single item self-report-80% usage within past week considered adherent	Modified General Assessment of Side-effects Scale (GASE), HADS
Pinheiro et al. (2017) [67]	USA	Clinical practice	Both	Not specified	1599 (1114)	Age: LP1 (4% < 35, 47% 35–50, 33% 50–64, 15% 65 or over) LP2 (3% < 35, 46% 35–50, 33% 50–64, 18% 60 or over), LP3 (3% < 35, 35% 35–50, 37% 50–64, 24% 65 or over), LP4 (2% < 35, 31% 35–50, 37% 50–64, 29% 65 or over) Ethnicity: LP1 (52%; Non-Hispanic white, 48% Non-Hispanic black) LP2 (50% Non-Hispanic white, 50% Non-Hispanic black) LP3 (68% Non-Hispanic white,32% Non-Hispanic black) LP4 (54% Non-Hispanic white, 46% Non-Hispanic black = 46%)	HER receptor positive Stage: LP1 (stage1 = 45%; stage 2 = 41%; stage 3 = 13%) LP2 (stage 1 = 37%; stage 2 = 46%; stage 3 = 17%) LP3 (stage 1 = 61%; Stage 2 = 33%; stage 3 = 7%); LP4 (stage 1 = 62%; stage 2 = 31%; stage 3 = 7%) Grade: LP1 (well-differentiated = 25%; moderately differentiated = 44%; Poorly differentiated or unknown = 31%) LP2 (well-differentiated = 18%; moderately differentiated = 45%; Poorly differentiated or unknown = 37%) LP3 (well-differentiated = 34%; moderately differentiated = 48%; Poorly differentiated = 18%) LP4 (well-differentiated = 27%; moderately differentiated = 48%; Poorly differentiated or unknown = 25%)	Adherence	Self-report questions and modified MMAS, adherent if missed ≤2 pills within past 2 weeks	FACT-B
Quinn et al. (2016) [68]	Ireland	Oncology clinic	Both	Tamoxifen = 62%, AI = 32.2%, unknown = 5.8%	261 (255)	Age: *M* = 57.88 (±9.1)	HER receptor positive	Persistence	MMAS-8, temporary discontinuation less than 6 months, permanent discontinuation more than 6 months	Unclear
Schover et al. (2014) [69]	USA	Clinical practice	AI	Not specified	296 (129)	Age: Adherent *M* = 63.3 (*SD* = 8.7) Non-adherent *M* = 64.5 (*SD* = 8.9) Ethnicity: Adherent: 80.4% White, not Hispanic, 7.8% Hispanic, 9.8% African-American, 2% Asian Pacific Islander Non-adherent: 85% White, not Hispanic, 10% Hispanic, 0% African-American, 5% Asian Pacific Islander	HER receptor positive	Adherence	3-item Adherence Estimator® developed by Merck	BCPT 8-symptom scale (BESS), Female Sexual Function Index, 10-item Menopausal Sexual Interest Questionnaire (MSIQ)
Shinn et al. (2019) [70]	USA	Cancer centre	Not specified		339 (216)	Age: Discontinued: *M* = 57.6 (*SD* = 7.8) Still taking: *M* = 57.8 (*SD* = 11.3) Ethnicity: Discontinued: 10.3%, Hispanic, 64.1% White, 15.4% African American, 7.7% Asian, 2.6% Other Still taking: 15.3% Hispanic, 64.8% White, 13.1% African American, 5.7% Asian, 1.1% Other	HER receptor negative Stage: Discontinued (DCIS = 7.7%, stage1 = 38.5%, stage 2 = 33.3%, stage 3 = 15.4%) still taking (DCIS = 11.2, stage 1 = 37.9%, stage 2 = 35.4%, stage 3 = 14.9%)	Adherence	Duration of adherence calculated by subtracting month and year of survey from month and year of diagnosis; > 80% self-reported usage considered adherent	Self-reported severity of adverse effects
Spencer et al. (2020) [71]	USA	Cancer centre	Both	AI (not specified) and Tamoxifen	2998 (1231)	Age: *M* = 53.19 (*SD* = 10.91) Ethnicity: 57.6% Non-black, 42.4% African-American	HER receptor positive Stage: stage1 = 49%, stage 2 = 36.6%, stage3 = 12.7%, stage unknown = 1.7%	Adherence	Self-reported survey	Discontinuation due to side effects
Stahlschmidt et al. (2019) [72]	Brazil	Hospital	Both	Not specified	58 [58]	Age: T: *M* = 59 (*SD* = 12) AI: *M* = 56, (*SD* = 11)	HR receptor positive T (stage 0: n = 1, stage1: n = 16, stage 2:n = 14, stage3/4:n = 11) AI (stage0: n = 0, stage1: n = 2, stage2:n = 6, stage 3/4:n = 8)	Adherence	MMAS-4	EORTC QLQ & BR23, 23 additional items
Stahlschmidt et al. (2020) [73]	Brazil	Hospital	Both	Tamoxifen, Anastrozole and Exemestane	58 [58]	Age: Tamoxifen: *M* = 59 (*SD* = 12), AI: *M* = 56 (*SD* = 11) Ethnicity: T: 78% Caucasian (*N* = 33) 22% Non-Caucasian (*N* = 9) AI: 94% Caucasian (*N* = 15), 22% Non- Caucasian (*N* = 9)	Stage 0-II: T: 31 (74%); AI: 8 (50%) Stage III-IV: T: 11 (26%); AI: 8 (50%)	Adherence	MMAS-4	International Consultation on Incontinence Questionnaire (ICIQ) Overactive Bladder (OAB)
Stanton et al. (2014) [74]	USA	Clinical practice	Both	Tamoxifen, Anastrazole, Exemestane and Letrozole	1465 (1371)	Age: *M* = 56.03, (*SD* = 8.72, Range = 25–86) Ethnicity: 94% Non-Hispanic White, 2% African American, 1% Asian American, 2% Latina	HR Positive Stage 0 = 4%, Stage1 = 37%, Stage2 = 37%, Stage3 = 12%, Stage 4 = 9%, Don't Know = 5%	Both	Adapted MMAS	HADS, BCPT symptom checklist, self-report physical symptoms
Tan et al. (2015) [75]	USA	Clinical practice	Both	AI (not specified) and Tamoxifen	428 (428)	Age at diagnosis: 8.2% < 65, 36.2% 65–74, 43.7% 75–84, 11.9% 85 or over	HER receptor positive stage1 = 55.8%, stage2 = 34.8%, stage3 = 9.4%	Both	MPR-80% adherence cut-off	Use of medications that treated known side effects (medical records)
Wagner et al. (2018) [76]	Canada	Hospital, cancer centre	AI	Anastrazole and Exemestane	688 (686)	Age: Anastrazole arm: *M* = 65.9 (*SD* = 9.4, Median = 65.7, Range = 32.3–89.8) Exemestane arm: *M* = 65.4 (*SD* = 9, Median = 64.7, Range = 43.1–99.8) Ethnicity: A arm: 96% white, 2.7% Black, 1.3% other. E arm: 96.2% white, 3.8% black, 0% other	HER positive Stage: Pathologic T stage: A arm (T1 = 74.9%, T2 = 22.9%, T3 = 2.2%) E arm (T1 = 76.2%, T2 = 22.5%, T3 = 1%, Tx = 0.3%)	Both	Discontinued within 5 years	Aggregate score for items from FACT-ES & FACT-G
Walker et al. (2016) [77]	USA	Hospital	Both	AI (not specified) and Tamoxifen	106 [82]	Age: *M* = 38.5(*SD* = 4.1) Ethnicity: Non-white (*N* = 10), white (*N* = 94), missing (*N* = 2)	HER receptor positive stage0 = 3, stage1 = 32, stage2 = 37, stage3 = 21, stage4 = 8, unsure = 2, missing = 3	Adherence	Adapted MMAS	BCPT Symptom checklist
Wheeler et al. (2019) [87]	USA	Cancer centre	Both	Tamoxifen and AI (not specified)	2015 (1280)	Age: <45 (*N* = 289), 45–54 (*N* = 422), 55–64 (*N* = 328), >65 (*N* = 241)	Stage: At diagnosis: 637 were stage 1, 462 were stage 2, 159 were stage 3, 22 unknown	Adherence	Self-reported usage (<80% usage within past 2 weeks)	FACT-B, FACT-ES
Wouters et al. (2014) [78]	Netherlands	Hospital, pharmacy, patient organization	Both	Tamoxifen and AI (not specified)	241 (241)	Age: M = 57(*SD* = 10)	Not reported	Adherence	MARS and MMAS-8: created an intentional non-intentional adherence score by adding together relevant questions	Tailored Medicine Inventory (TMI)
Wuensch et al. (2015) [79]	Germany	Clinical practice	Not specified		523 (281)	Age: Median = 51	HER positive	Both	Unclear	Self-report number and intensity and side effects
Xu et al. (2020) [80]	China	University hospital	Both	AI and Tamoxifen	1875 (888)	Age: Median: 54 (Range = 47–62) Ethnicity: 92.7% Han (*N* = 823), 7.3% Minorities (*N* = 65)	HER2 Negative 785 (88.4%) HER2 Positive 103 (11.6%)	Both	MMAS-4	Number of side effects
Yi et al. (2018) [81]	South Korea	University hospital	Both	Tamoxifen, AI, T and Zoladex, others	110 (110)	Age: *M* = 53.56 (Range = 38–69)	Stage: 51% stage 0 or 1, 36% stage 2 Grade: 2	Adherence	Unclear	BPI, MRS
Yin et al. (2018) [82]	USA	University hospital	Both	AI only, SERM only, AI and SERM	1106 (1106)	Age at diagnosis: *M* = 53.9 (*SD* = 11.1) Ethnicity: 91.3% White, 5.9% African American, 1.9% Asian, 0.9% Other	12.9% stage 1, 87.1% stages 1/2 Grade: 1 or 2	Persistence	Discontinued within 5 years	Mentions of side effects in online discussion forum
Ziller et al. (2009) [83]	Germany	Hospital	Both	Tamoxifen and Anastrazole	100 [89]	Age: T (*M* = 65), Ana (*M* = 72)	Stage: T (20% in situ, 60%T1, 18%T2, 0%T3, 2%T4 Ana (in situ = 3%, T1 = 72%, T2 = 20%, T3 = 0%, T4 = 8%)	Adherence	MPR and self-report (80% cut-off)	MRS and Global Quality of Life Scale

### 3.3 Quality assessment

#### 3.3.1 Most observational and cross-sectional studies were rated good quality (n = 43), whereas most controlled intervention studies were rated fair quality (n = 6). Three studies (1 controlled and 2 observational studies) were rated poor quality

(see Table 2, Table 3).

**Table 2:** Quality Assessment of Observational and Cross-Sectional Studies (NHLBI quality assessment tool).

Study Reference	Quality Rating	Q1	Q2	Q3	Q4	Q5	Q6	Q7	Q8	Q9	Q10	Q11	Q12	Q13	Q14	Q15
Bedi et al. (2020)	Fair	Y	Y	NA	Y	N	N	Y	NA	Y	N	NA	CD	NA	Y	N
Bowles et al. (2012)	Good	Y	Y	Y	Y	N	NA	Y	N	Y	N	Y	N	Y	Y	Y
Brett et al. (2018)	Good	Y	Y	Y	Y	N	N	Y	Y	Y	N	Y	N	Y	Y	Y
Brier (2018)	Good	Y	Y	Y	Y	Y	Y	Y	Y	Y	N	Y	NR	NR	Y	Y
Brier et al. (2015)	Good	Y	Y	Y	Y	Y	Y	Y	Y	Y	N	Y	N	NA	Y	Y
Brier et al. (2017)	Good	Y	Y	CD	Y	Y	NA	Y	Y	Y	N	Y	N	NA	Y	Y
Brier et al. (2018)	Good	Y	Y	CD	Y	Y	N	Y	Y	Y	N	N	N	NA	Y	Y
Bright et al. (2016)	Good	Y	Y	Y	Y	N	N	Y	Y	Y	N	Y	N	NA	Y	Y
Chim et al. (2013)	Good	Y	Y	Y	Y	N	Y	Y	Y	Y	Y	Y	N	Y	Y	Y
Cluze et al. (2012)	Good	Y	Y	Y	Y	N	Y	Y	NA	Y	Y	Y	Y	Y	Y	N
Corter et al. (2018)	Good	Y	Y	Y	Y	N	Y	Y	Y	Y	Y	Y	N	Y	Y	Y
Demissie et al. (2001)	Good	Y	Y	Y	Y	N	Y	Y	N	Y	Y	Y	N	Y	Y	N
Font et al. (2019)	Good	Y	Y	Y	Y	N	Y	Y	Y	Y	Y	Y	N	Y	Y	Y
Gao et al. (2018)	Good	Y	Y	Y	Y	N	Y	Y	Y	Y	Y	Y	N	Y	Y	Y
Grossman (2016)	Good	Y	Y	Y	Y	N	Y	Y	NA	Y	Y	N	CD	Y	Y	Y
Hadji et al. (2014)	Good	Y	Y	Y	Y	N	Y	Y	Y	Y	Y	Y	N	Y	Y	Y
Helland et al. (2019)	Good	Y	Y	Y	Y	N	Y	Y	Y	Y	Y	Y	N	Y	Y	Y
Henry et al. (2010)	Good	Y	Y	Y	Y	N	Y	Y	Y	Y	Y	Y	N	Y	Y	N
Henry et al. (2012)	Good	Y	Y	N	Y	N	Y	Y	Y	Y	Y	Y	N	Y	Y	Y
Henry et al. (2017)	Good	Y	Y	Y	Y	N	Y	Y	Y	Y	Y	Y	N	Y	Y	Y
Hershman et al. (2016)	Good	Y	Y	Y	Y	N	Y	Y	Y	Y	Y	Y	N	Y	Y	Y
Hsieh et al. (2015)	Good	Y	Y	CD	Y	N	N	Y	Y	Y	N	Y	N	Y	Y	N
Iacorossi et al. (2016)	Good	Y	Y	NR	Y	Y	NA	NA	Y	Y	Y	Y	N	Y	Y	Y
Kahn et al. (2007)	Good	Y	Y	Y	Y	N	Y	Y	Y	Y	Y	Y	N	Y	N	N
Kimmick et al. (2017)	Fair	Y	Y	Y	Y	N	NA	NA	NA	Y	N	Y	N	NA	Y	Y
Kostev et al. (2013)	Good	Y	Y	Y	Y	Y	Y	Y	Y	Y	Y	Y	N	N	Y	Y
Lash et al. (2006)	Good	N	Y	Y	Y	Y	Y	Y	Y	Y	Y	Y	N	N	Y	N
Liu et al. (2013)	Good	N	Y	Y	Y	N	N	Y	Y	Y	Y	Y	N	N	Y	N
Llarena et al. (2015)	Good	Y	Y	Y	Y	N	Y	Y	Y	Y	Y	Y	Y	Y	Y	Y
Markovitz et al. (2017)	Good	Y	Y	Y	Y	N	NA	Y	Y	Y	N	Y	N	NA	Y	Y
Mao et al. (2020)	Good	Y	Y	Y	Y	N	Y	Y	Y	Y	Y	Y	NA	NA	N	Y
Moon et al. (2019)	Good	Y	Y	Y	Y	N	NA	Y	Y	Y	Y	Y	N	Y	Y	Y
Nabieva et al. (2018)	Good	Y	Y	Y	Y	N	NA	Y	Y	Y	Y	Y	N	N	Y	Y
Nestoriuc et al. (2016)	Good	Y	Y	Y	Y	Y	NA	Y	Y	Y	Y	Y	Y	Y	Y	Y
Pan et al. (2018)	Good	Y	Y	Y	Y	Y	Y	Y	Y	Y	N	Y	NA	Y	Y	Y
Pinheiro (2017)	Good	Y	Y	Y	Y	N	Y	Y	NA	Y	N	Y	NA	N	Y	Y
Quinn (2016)	Fair	Y	Y	Y	Y	N	N	Y	Y	N	N	Y	NA	NA	Y	Y
Schover (2014)	Fair	Y	Y	N	Y	N	N	Y	Y	Y	N	Y	NA	NA	N	Y
Shinn (2019)	Good	Y	N	Y	Y	Y	Y	Y	Y	Y	N	Y	NA	NA	Y	Y
Spencer (2020)	Fair	Y	Y	N	Y	N	Y	Y	N	N	N	N	NA	NA	Y	N
Stahlschmidt (2019)	Fair	Y	N	Y	Y	N	N	Y	Y	Y	N	Y	NA	NA	Y	Y
Stahlschmidt et al. (2020)	Poor	Y	Y	CD	Y	N	CD	CD	N	N	N	Y	NA	NA	Y	Y
Stanton (2014)	Good	Y	Y	Y	Y	Y	N	Y	Y	Y	N	Y	NA	NA	Y	Y
Tan (2015)	Good	Y	Y	Y	Y	Y	Y	Y	NA	Y	N	Y	NA	Y	Y	Y
Wagner (2018)	Good	Y	Y	Y	Y	Y	Y	Y	Y	Y	Y	Y	NA	Y	Y	Y
Walker (2016)	Fair	Y	Y	Y	N	N	N	Y	NA	Y	N	Y	NA	NA	N	Y
Wheeler (2019)	Good	Y	Y	Y	Y	Y	Y	Y	Y	N	Y	Y	NA	Y	Y	Y
Wouters (2014)	Good	Y	y	Y	Y	N	N	Y	Y	Y	N	Y	NA	NA	Y	Y
Wuensch (2015)	Poor	Y	N	NA	Y	N	N	Y	N	N	N	N	NA	NA	N	N
Xu et al. (2020)	Good	Y	Y	Y	Y	Y	NA	Y	N	Y	Y	Y	N	Y	N	Y
Yi (2018)	Fair	Y	Y	NA	Y	N	N	Y	Y	Y	N	N	NA	NA	N	N
Yin (2018)	Good	Y	Y	NA	Y	Y	N	Y	Y	Y	Y	Y	NA	NA	Y	Y
Ziller (2009)	Good	Y	Y	Y	Y	Y	N	Y	Y	Y	N	Y	NA	NA	N	Y

Note A - Question items: Q1 Was the research question or objective in this paper clearly stated? Q2 Was the study population clearly specified and defined? Q3 Was the participation rate of eligible persons at least 50%? Q4 Were all the subjects selected or recruited from the same or similar populations (including the same time period)? Were inclusion and exclusion criteria for being in the study pre-specified and applied uniformly to all participants? Q5 Was a sample size justification, power description, or variance and effect estimates provided? Q6 For the analyses in this paper, were the exposure(s) of interest measured prior to the outcome(s) being measured? Q7 Was the timeframe sufficient so that one could reasonably expect to see an association between exposure and outcome if it existed? Q8 For exposures that can vary in amount or level, did the study examine different levels of the exposure as related to the outcome (e.g., categories of exposure, or exposure measured as continuous variable)? Q9 Were the exposure measures (independent variables) clearly defined, valid, reliable, and implemented consistently across all study participants? Q10 Was the exposure(s) assessed more than once over time? Q11 Were the outcome measures (dependent variables) clearly defined, valid, reliable, and implemented consistently across all study participants? Q12 Were the outcome assessors blinded to the exposure status of participants? Q13 Was loss to follow-up after baseline 20% or less? Q14 Were key potential confounding variables measured and adjusted statistically for their impact on the relationship between exposure(s) and outcome(s)? Q15 Was adherence/persistence measured appropriately and clearly described? Note B - Key Y = yes; N = No; CD = cannot determine; NA = not applicable; NR = not reported.

**Table 3:** Quality Assessment of Controlled Intervention Studies (NHLBI quality assessment tool).

Reference	Quality Rating	Q1	Q2	Q3	Q4	Q5	Q6	Q7	Q8	Q9	Q10	Q11	Q12	Q13	Q14	Q15
Bender et al. (2014)*	Good	Y	Y	Y	Y	Y	Y	Y	NA	CD	N	Y	NA			Y
Cuzick (2007)	Poor	N	NR	NR	NR	NR	Y	CD	CD	CD	CD	Y	N	N	Y	N
Henry et al. (2013)	Fair	Y	Y	CD	CD	CD	Y	N	Y	Y	CD	Y	N	Y	N	N
Jackisch et al. (2019)	Fair	Y	NR	NR	NR	NR	Y	N	NR	Y	NR	Y	N	Y	NR	Y
Kadakia et al. (2016)	Fair	Y	NR	NR	NR	NR	Y	N	Y	Y	Y	Y	N	Y	NR	N
Kidwell et al. (2014)	Fair	Y	NR	NR	NR	NR	Y	Y	Y	Y	Y	NR	N	Y	NR	N
Kool et al. (2014)	Fair	Y	NR	N	NR	NR	Y	N	NR	NR	NR	Y	Y	Y	NR	N
Kyvernitakis et al. (2014)	Fair	Y	NR	N	Y	N	Y	N	NR	NR	NR	Y	N	Y	NR	Y
Li et al. (2019)	Good	Y	Y	NR	NR	Y	Y	Y	Y	Y	Y	Y	Y	Y	NR	N

Note A - Question items: Q1 Was the study described as randomized, a randomized trial, a randomized clinical trial, or an RCT? Q2 Was the method of randomization adequate (i.e., use of randomly generated assignment)? Q3 Was the treatment allocation concealed (so that assignments could not be predicted)? Q4 Were study participants and providers blinded to treatment group assignment? Q5 Were the people assessing the outcomes blinded to the participants' group assignments? Q6 Were the groups similar at baseline on important characteristics that could affect outcomes (e.g., demographics, risk factors, co-morbid conditions)? Q7 Was the overall drop-out rate from the study at endpoint 20% or lower of the number allocated to treatment? Q8 Was the differential drop-out rate (between treatment groups) at endpoint 15% points or lower? Q9 Was there high adherence to the intervention protocols for each treatment group? Q10 Were other interventions avoided or similar in the groups (e.g., similar background treatments)? Q11 Were outcomes assessed using valid and reliable measures, implemented consistently across all study participants? Q12 Did the authors report that the sample size was sufficiently large to be able to detect a difference in the main outcome between groups with at least 80% power? Q13 Were outcomes reported or subgroups analysed prespecified (i.e., identified before analyses were conducted)? Q14 Were outcomes reported or subgroups analysed pre-specified (i.e., identified before analyses were conducted)? Q15 Was adherence/persistence measured appropriately and clearly described? *Before and After Study.

Note B - Key Y = yes; N = No; CD = cannot determine; NA = not applicable; NR = not reported.

### 3.4 Operationalising adherence

#### 3.4.1 Measuring adherence

Most studies used self-report adherence measures. The most frequently used validated self-report measures were the Medication Adherence Rating Scale (MARS) [84], [4,31,49,78], the 8-item Morisky Medication Adherence Scale (MMAS-8) [85], [35,52,57], and the 4-item Morisky Medication Adherence Scale (MMAS-4) [86] [64,72,73,80]. Seven studies created their own adherence measure [38,53,58,67,69,70,87]. Nine studies used indirect adherence measures, including electronic monitoring devices, counting pills and medication chart reviews. Two studies [21,43] used a Medication Event Monitoring System (MEMS), an electronic device recording how often medication packaging is opened. Four studies used a Medication Possession Ratio (MPR), referring to the days during an observed time period where a person was in possession of their medication. This was calculated from data extracted from pharmacy records [41,45], Medicare claims [75], and hospital prescription records [83]. Two studies extracted relevant information from physician notes regarding appointments and phone calls in hospital medical charts [32,33]. No studies used a direct measurement of adherence (i.e., analysis of blood/urine).

#### 3.4.2 Defining adherence

##### 3.4.2.1 Self-report

Nine studies divided participants into ‘adherent’ and ‘non-adherent’ using self-report measures. One study used the MARS, where a score of 24 or above was considered adherent [4]. One study deemed participants adherent if they reported still taking HT at 36 months after initiation [61]. Seven studies established an adherence cut-off based on self-report of the proportion of medication taken. Two studies deemed participants adherent if they reported taking at least 80% of prescribed HT doses [53,70]. One study deemed participants to be adherent if they reported never having missed a dose during the previous 30 days [38], whereas one [58] defined participants adherent if they reported never having forgotten to take their medication. Two studies [67,87] considered participants adherent if they missed less than 3 pills over the previous 2 weeks. One study [15] considered participants adherent if they reported taking 80% of their medication over the previous week.

##### 3.4.2.2 Indirect measures

Studies using indirect measures of adherence classified participants as adherent or non-adherent based on data obtained from medical records, MPR, and MEMS. Three studies considered non-adherence as any interruption or premature discontinuation of treatment [[32], [33], [34]], using data extracted from medical records. Two studies used medical records to monitor self-reported tablet intake, required to be at least 80% to be considered adherent [20,53]. A MPR was used to define adherence in 3 studies [41,45,75]. These studies considered participants adherent if they were in possession of HT medication 80% of the time. One study required both an MPR of at least 80%, and self-report of taking at least 80% of the prescribed HT dose [83]. One study used a MEMS [43] to define adherence by using the device to assess whether participants took at least 80% of the prescribed medication doses. One study [44] required both patient and clinician report of 80% of HT medication being taken.

### 3.5 Distinguishing between intentional and unintentional non-adherence

#### 3.5.1 Defining and measuring intentional and unintentional non-adherence

In total, eight studies made a distinction between the measurement of intentional and unintentional non-adherence. All of these studies used self-report measures to make this assessment.

Four studies used the MARS to measure intentional and unintentional non-adherence. This measure includes 4 items measuring intentional non-adherence, and 1 measuring unintentional non-adherence. Two studies [4,49] used this measure alone. One study [31] used the MARS and 4 additional questions to measure more specific intentional and unintentional non-adherence behaviours. Another study [78] used the MARS, additional questions selected from the MMAS-8, and additional questions about forgetting to take HT medication.

Participants were classed as intentionally nonadherent based on a total score of 19 or below for relevant MARS questions [4], 3 or below for all MARS and additional intentional items [31], and less than 80% of the maximum total score for MARS and MMAS-8 intentional questions [78]. They were classed as unintentionally nonadherent based on a total score of 4 or below for relevant MARS questions [4], 3 or below for MARS item 1 and additional unintentional items [31], and less than 80% of the maximum total score for MARS and MMAS-8 unintentional questions [78].

One study [43] used the Pill Count Form (PCF) which included a question asking how often participants forgot to take their medication and how often they chose not to take it. One [57] used the MMAS-8 and 8 additional items, asking how often participants engaged in non-adherence behaviours. They then gave participants a score based on how many intentional and unintentional non-adherence behaviours they reported. One [88] used a combination of the MMAS-8 and a visual analog scale. Another study [71] used HT-specific questions selected from a range of existing self-report adherence measures.

### 3.6 Operationalising persistence

#### 3.6.1 Measuring persistence

Seven studies [19,30,40,42,54,55,74] measured persistence subjectively, by simply asking participants if they were still taking their HT medication. Eleven studies used indirect measures of persistence, including medical records [36,46,63,82] and pharmacy databases [29,37,45,50,51,59,62].

#### 3.6.2 Defining persistence

Five studies used self-report measures to classify participants as persistent or non-persistent. Participants were classified as persistent if they reported using HT for at least 5 years [42,89] or 4 years [55]. Two studies [40,74] classified participants as persistent if they reported still taking HT at the time of the study (irrespective of the duration of the prescription). Definitions of persistence based on indirect measures were based on data from medical records and pharmacy databases. For persistence data based on medical records, participants were considered persistent if medical records indicated they took HT for 5 years [19,82] or if they fulfilled the recommended treatment period [36,46]. One study [63] used medical records to identify gaps between prescriptions: a gap of 180 days or more between prescriptions was considered non-persistent. Pharmacy databases were used by 5 studies to classify participants as persistent or non-persistent. One study [62] classified participants non-persistent if pharmacy data indicated they had discontinued their medication within 5 years. Other studies classified participants as non-persistent based on gaps between prescription uptake of 60 days [37], 90 days [50,59] and 180 days [51]. Two studies considered both the timing of discontinuation and the gap between prescriptions. One [45] defined non-persistence as a gap between prescriptions of 60 days or more, occurring before the end of the recommended treatment duration. One [59] focused on a prescription gap of 90 days, within 3 years of HT initiation.

### 3.7 Frequency of measured HT side effects

A wide range of HT side effects were measured across all studies (see Table 4). The most frequently measured were musculoskeletal or joint pain (*N* = 22), mood disturbance/depression (*N* = 22), hot flashes (*N* = 15), sleep problems/insomnia (*N* = 12), anxiety/nervousness (*N* = 12), fatigue/tiredness (*N* = 9), weight gain (*N* = 9), and concentration/memory problems (*N* = 8). The remaining side effects were reported by 7 (or fewer) studies. This review focuses on these most commonly measured side effects.

**Table 4:** Side effects measured in included studies.

Side Effects	N Studies Reported	Measures			Association with adherence/persistence		
		SR^a^ questionnaire	Symptom checklist	Medical records	-ve^b^	c+ve	none
Not Specified Side Effects	24	17	4	3	16	1	7
Mood disturbances/Depression	22	16	5	1	12	1	9
Pain (Musculoskeletal, Joint or Muscular)	22	19	2	1	11		11
Hot Flashes	15	13	1	1	2		13
Anxiety/Nervousness	12	4	6	2	6	1	5
Sleep problems/Insomnia	12	11		1	4	1	7
Fatigue/Tiredness	9	6	3		2		7
Weight Gain	9	9			3		6
Concentration/Memory Problems	8	4	4		6		2
Vaginal Bleeding	7	6	1		2		5
Vaginal Dryness	7	7			3		4
Arthralgia	6	4	2		3		3
Bladder problems/Incontinence	6	5	1		1		5
Loss of Libido	6	6			1		5
Nausea/Vomiting	6	5	1		4		2
Appetite Loss	5	3	2		2		3
Gastrointestinal Problems (Bloating, Indigestion, Heartburn)	5	3	2		1		4
Night Sweats	5	4	1			1	4
Pain (not specified)	5	3		2	2	1	2
Vision Problems	5	4	1		2		3
Menopausal Symptoms	4	4				1	3
Sexual Problems	4	4					4
Shortness of Breath	4	3	1		1		3
Breast Tenderness	3	2	1				3
Diarrhoea	3	2	1		1		2
Hair loss/Alopecia	3	3			1		2
Headache	3	2	1		2		1
Lymphoedema/Fluid retention	3	3			1		2
Pain during Intercourse	3	3					3
Bone Fracture	2	2					2
Constipation	2	2					2
Gynaecological Problems	2	1	1		1		1
Vaginal Discharge	2	2					2
Bone loss/Osteoporosis	1	1					1
Dizziness	1	1					1
Heart Discomfort	1	1			1		

a Self-reported.

b Negative association.

c Positive association.

### 3.8 Measurement of side effects

Most studies used self-report to measure specific side effects of HT. The most frequently used validated measures were the Breast Cancer Prevention Trial (BCPT) symptom checklist [21,38,54,56,74,77,92], Centre for Epidemiologic Studies–Depression (CES-D) [37,43,54,56,64], Hospital Anxiety and Depression Scale (HADS) [15,32,34,56,66,74,88] and the Brief Pain Inventory (BPI) [[32], [33], [34],^36^,^57^,^81^]. Three studies used the proxy measure of pharmacy records of prescriptions for the management of HT side effects [29,51,75]. Two studies used medical records of dropout due to HT adverse effects [48,65], 1 study used medical records of diagnosed issues linked to side effects [59], and 2 used medical records of side effects [61,63]. Twenty-four studies reported that patients reported HT side effects but did not specify what the side effects were.

### 3.9 Relationship between side effects and adherence/persistence

**Fig. 2:**
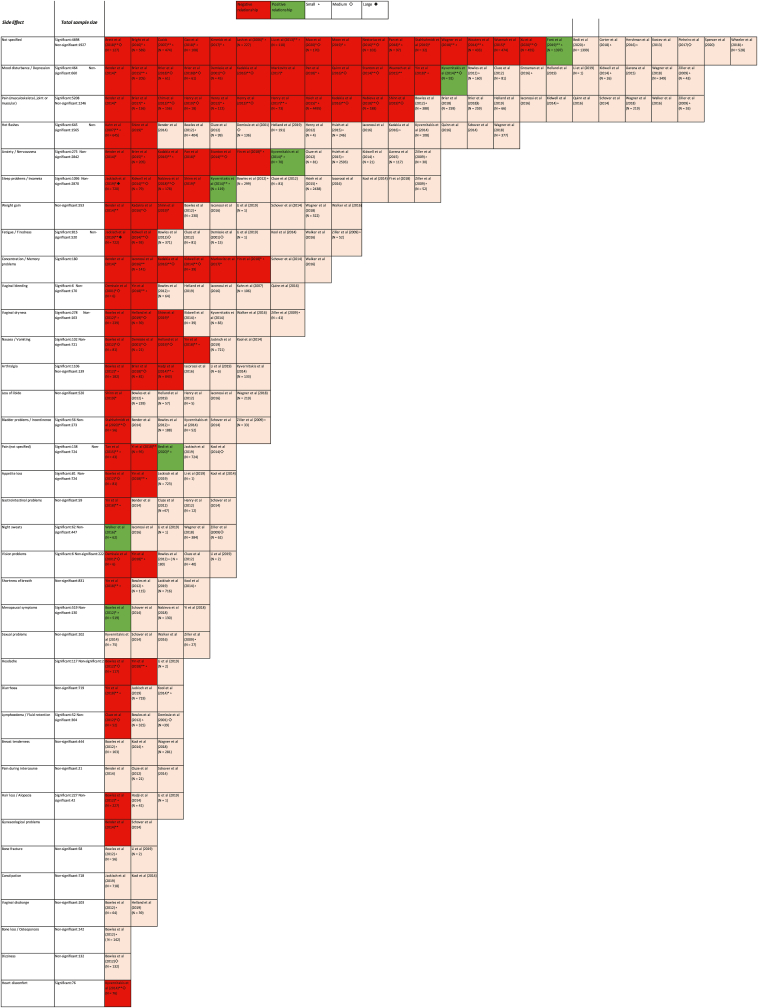
Harvest plot.

The Harvest Plot (Fig. 2) presents a full list of HT side effects reported across all 62 studies. It also indicates the studies that reported a relationship (positive or negative) between a HT side effect and adherence/persistence, and those studies that reported no relationship.

### 3.10 Relationship between side effects and intentional/unintentional adherence

Only 2 studies distinguished between intentional and unintentional adherence when investigating the potential influence of side effects. Both were rated ‘good’ quality. One study [43] found that depression was not significantly related to either intentional or unintentional adherence, whereas another [88] found that depression was significantly negatively related to unintentional and intentional non-adherence. This study also found a significant negative relationship between anxiety and intentional non-adherence, but not between anxiety and unintentional non-adherence.

The relationships between the 8 most frequently measured side effects and adherence/persistence are evaluated below.

### 3.11 Musculoskeletal/joint pain

The relationship between musculoskeletal/joint pain on adherence was evaluated in 12 studies, all rated as good/fair quality. Six [21,33,36,49,65,70] found a significant negative relationship and the other 6 found no significant relationship [32,34,52,69,77,83]. Ten studies investigated the influence of musculoskeletal/joint pain on persistence, all rated as good/fair quality. Five found a significant negative relationship [[46], [47], [48],^51^,^54^] and the other 5 found no significant relationship [30,45,56,68,76]. Across these studies, the total sample size reporting a significant negative relationship was much higher (5208) than those reporting no significant relationship (1246), indicating that the existence of a significant negative relationship between musculoskeletal/joint pain and adherence and persistence seems to be supported by a more substantial body of research, all of good or fair quality.

### 3.12 Mood disturbance/depression

Eleven studies investigated the relationship between mood disturbance/depression and adherence to HT. The majority of these reported a significant negative relationship [15,21,32,34,64,88], of which, 6 were rated good quality and 1 [79] was rated poor quality. One fair quality study [20] reported a significant positive relationship with adherence and 3 good quality studies [43,74,83] found no significant relationship. The relationship between mood disturbance/depression and HT persistence was explored in 12 studies. Five good/fair quality studies [40,54,68,74,82] identified a significant negative relationship. Six good/fair quality studies reported no significant relationship [30,37,45,56,62,76]. Across these studies, the total sample size of studies reporting no significant relationship between mood disturbance/depression and adherence/persistence (660) was slightly higher than those reporting a significant relationship (464). Overall, the relationship between mood disturbance/depression and adherence/persistence to HT remains very unclear from the existing literature.

### 3.13 Hot flashes

Five studies investigated the relationship between hot flashes and HT adherence. Only one of these [70], rated good quality, found a significant negative relationship with adherence. The other 4 studies [20,21,52,69], rated good/fair quality, found no significant relationship. Ten studies investigated the relationship between hot flashes and HT persistence. Only one [55] good quality study identified a significant negative relationship. The remaining 9 good/fair quality studies found no significant relationship [30,37,40,45,46,51,54,68,76]. The total sample size for those studies finding no significant relationship was 1565 compared to 645 who did report a significant relationship, indicating that the lack of a significant relationship between hot flashes and adherence/persistence to HT seems to be a more robust finding.

### 3.14 Sleep problems/insomnia

Eight papers investigated the relationship between sleep problems/insomnia and HT adherence. Three good/fair quality papers identified a significant negative relationship [53,65,70], 1 fair quality paper [20] found a significant positive relationship and the remaining 4 good/fair quality studies did not identify a significant relationship [52,58,81,83]. These findings make it difficult to draw any meaningful conclusions about the relationship between sleep problems/insomnia and HT adherence. Four studies investigated the relationship between sleep problems/insomnia and HT persistence. Only 1 fair quality study [56] found a significant negative relationship, whereas 3 good quality studies [30,37,51] found no significant relationship. Those studies finding no significant relationship had a much larger total sample size (2870) compared to the study reporting a significant relationship (1096). Overall, a higher volume of good quality studies suggest that no significant relationship exists between sleep problems and HT persistence.

### 3.15 Anxiety/nervousness

Six papers investigated the relationship between anxiety/nervousness and HT adherence. Three good quality studies [15,21,88] reported a significant negative relationship, 1 fair quality study [20] reported a significant positive relationship and 2 good quality studies [74,83] did not find any significant relationship. Seven studies investigated the relationship between anxiety/nervousness and HT persistence. Three good/fair quality studies found a significant negative relationship [54,74,82] and 4 good/fair quality studies found no significant relationship [37,51,56,62]. The total sample size for studies reporting a significant relationship between anxiety/nervousness and adherence/persistence (275) was much smaller than those finding no significant relationship (2842). However, the relationship between anxiety/nervousness and adherence/persistence remains very unclear from the available literature.

### 3.16 Fatigue/tiredness

Four studies investigated the relationship between fatigue/tiredness and HT adherence. One fair quality study [53] reported a significant negative relationship, whereas the other 3 studies (rated good/fair quality) found no significant relationship [58,77,83]. Five studies investigated the relationship between fatigue/tiredness and HT persistence. One fair quality study [56] found a significant negative relationship. However, 4 good quality studies [30,37,40,60] found no significant relationship. The total sample size of papers finding a significant relationship (815) was higher than those finding no significant relationship (520). However, the majority of studies investigating fatigue/tiredness found no significant relationship with adherence. Studies investigating the influence of fatigue/tiredness on persistence also seemed to suggest that there was no significant relationship. However, with so few studies exploring this side effect, further research is needed to aid our understanding of its impact on HT adherence/persistence.

### 3.17 Weight gain

Five studies investigated the relationship between weight gain and HT adherence. Two good quality studies [21,70] found a significant negative relationship, whereas 3 good/fair quality studies found no significant relationship [52,69,77]. Four studies investigated the relationship between weight gain and HT persistence. One fair quality study [54] found a significant negative relationship, and 3 good quality studies [30,60,76] found no significant relationship. The available research indicates that the relationship between weight gain and adherence is unclear and that there may be no significant relationship between weight gain and HT persistence. However, it is difficult to draw any meaningful conclusions about the influence of weight gain on adherence/persistence to HT given the limited literature comprehensively exploring this relationship.

### 3.18 Concentration/memory problems

Five studies investigated the relationship between concentration/memory problems and HT adherence. Three good quality studies [21,52,64] found a significant negative relationship, and 2 fair quality studies [69,77] found no significant relationship. Three studies [54,56,82] investigated the relationship between concentration/memory problems and HT persistence. All 3 were rated good/fair quality and reported a significant negative relationship. The indication from these studies is that there may be a significant negative relationship between concentration/memory problems and HT adherence/persistence. However, further research is needed to explore the impact of this side effect in more detail.

## 4 Discussion

Despite the importance of HT for reducing the risk of breast cancer recurrence [2,3], research indicates that suboptimal adherence and non-persistence are a threat to the success of this treatment [4,5,94]. Understanding the factors influencing adherence and persistence behaviours is therefore important for improving long-term outcomes in breast cancer survivors. The aim of this review was to identify, evaluate and summarise the relationship between HT side effects and patterns of adherence and persistence. For clarity, we focused on evaluating the relationship between the 8 most commonly-measured side effects in studies exploring adherence and persistence to hormone therapy: musculoskeletal/joint pain, mood disturbance/depression, hot flashes, sleep problems/insomnia, anxiety/nervousness, weight gain, fatigue, and concentration/memory problems.

### 4.1 Overall comment on findings

We set out to capture the impact of individual side effects on the magnitude of adherence and persistence to HT. This review identified a lack of consistency in the measurement of adherence and the definition of persistence across studies. The instruments used to measure side effects also varied significantly. This variation and lack of consistency makes it difficult to evaluate and summarise the degree of adherence and persistence across studies. Wide-ranging sample sizes, variation in the menopausal status of women included in studies and variation in HT drugs, also prevents effective comparison across studies. Taken together, these factors make it challenging to draw clear conclusions about the impact of individual side effects from the available literature.

### 4.2 Relationship between side effects and adherence

Based on previous research [8,15,16], it was expected that the greater the experience of HT side effects, the poorer HT adherence and persistence would be. However, our review of the research does not identify a consistent relationship between HT side effects and adherence or persistence. This is similar to findings in a previous review by Moon et al. (2017) [8], who also failed to identify consistent relationships between side effects and adherence/persistence. The current review highlights the specific variation in study characteristics, which may contribute to this inconsistency, and evaluates the quality of research exploring the relationship between side effects and HT adherence/persistence.

The majority of studies that found a significant relationship between side effects and adherence/persistence, found this relationship to be in a negative direction. Only one study [20] found a significant positive relationship between side effects (anxiety/nervousness, sleep problems/insomnia, and mood disturbance/depression) and HT adherence. For several side effects (sleep problems/insomnia, weight gain, joint/musculoskeletal pain, anxiety) the number of good quality studies identified in support of their influence on adherence/persistence did not differ from the number of studies finding no significant relationship. This prevents us from drawing strong conclusions about the influence of these side effects. Furthermore, a low number of studies investigating particular side effects (memory/concentration issues, weight gain, fatigue) prevents effective evaluation of the relationship between these side effects and HT adherence/persistence.

The only side effects where relationships with HT adherence/persistence could be clearly evaluated were mood disturbance/depression, and hot flashes. Over twice as many good quality papers identified a significant negative relationship between mood disturbance/depression and adherence, in comparison to those finding a significant positive relationship or no significant relationship. For hot flashes, a much higher number of good quality studies found no significant relationship between HT adherence and persistence, indicating hot flashes do not seem to have an impact.

### 4.3 Measurement of HT side effects

The majority of studies used validated self-report side effect measures, such as the HADS, BPI and BCPT Symptom checklist. However, some used proxy measures such as medical records and prescription records. These types of measures do not capture the potential range in severity of side effects, side effects patients may not have informed their doctor about, or those that cannot be managed successfully by medication. The variation in instruments used to measure side effects was also identified in a scoping review by Zhu and colleagues [23] as a barrier to synthesising data on the influence of side effects. The current review indicates that variation in the measurement of HT side effects remains an obstacle to our understanding of HT adherence and persistence.

### 4.4 Adherence

All studies identified in this review used indirect measures of adherence. The most frequent method of measurement was self-report. Although some studies utilised reliable, valid self-report measures such as the MARS, MMAS-8 and MMAS-4, many developed their own measure, resulting in variation in the definition of ‘adherent’ vs ‘non-adherent’. Furthermore, self-report measures are susceptible to over-estimation of adherence levels due to social desirability and memory biases [90]. The potential impact of memory bias on self-reported adherence is important given the prevalence of cognitive impairment among breast cancer survivors [91]. This means they may be at particular risk of forgetting to take daily medication such as HT, however only 2 studies distinguished between intentional and unintentional non-adherence in relation to specific side effects. Understanding the potential difficulties that lead to unintentional non-adherence may be important in helping clinicians understand why their patients struggle to adhere. Our recent qualitative review (Peddie et al., 2021) [25] found that a supportive relationship with clinicians helps people feel they can seek help for their side effects, and therefore may facilitate adherence and persistence. Understanding the factors which make adherence difficult may help to improve patient-provider relations, and therefore lead to improvements in adherence and persistence in future.

In addition to measurement, the definition of adherence varied across studies. Studies using established measures applied consistent cut-off scores for self-report measures, and the standard 80% criteria was applied to MEMS and MPR measures. However, those that developed their own measure showed little consistency in their definition of adherence. This prevents us from effectively comparing the effect of side effects on adherence across studies, and also from gaining a clear idea of the prevalence of HT adherence. Variation in definition of adherence may also contribute to the wide-ranging prevalence estimates reported [14]. The limitations of frequently used adherence measures, and inconsistencies in the operationalisation and measurement of adherence, were also identified in previous reviews [8,22].

### 4.5 Persistence

The majority of studies measured persistence based on how many participants discontinued HT within a set time period. However, this time period varied widely across studies, ranging from 1 to 5 years. This makes comparison of persistence rates between studies difficult, inhibiting the ability to identify side effects which have the most profound impact on persistence. As HT can be prescribed for up to 10 years [94], measuring the number of people who discontinue within 1–5 years may not demonstrate how side effects can affect persistence for the full course of treatment.

Several studies defined non-persistence as a length of time between filling HT prescriptions. This may not accurately reflect non-persistence, as some people take a break from HT before returning to either the same type of medication or switching to a different type of HT [92,93]. A gap between prescriptions does not necessarily represent non-adherence behaviour and may be based on clinician recommendation. The importance of these management strategies, such as taking a clinician-approved break and switching to a different drug, have been highlighted in qualitative literature [25]. Variation in measurement and definition of persistence has been previously highlighted in Moon's (2017) [8] review. However the current review indicates that these limitations are still present in the existing research.

A lack of clarity regarding the difference between adherence and persistence in some studies makes it difficult to understand how side effects impact them separately. This distinction is important as side effects may impact adherence vs persistence differently. Importantly, the distinction between measurement of adherence and persistence was not always clear. One study defined adherence as HT use 36 months after initiation [61], which, in other studies, would have been used as a definition of persistence.

The majority of studies did not distinguish between the different types of HT. This means we cannot identify side effects specific to Tamoxifen or AIs, and therefore limits comparison across studies as these drugs can demonstrate different levels of toxicity [94].

### 4.6 Recommendation for future studies

Future research would benefit from using a consistent definition of adherence, such as the definition proposed by Wassermann and Rosenberg (2017) [7]. Research would also benefit from implementing the same method of measurement (which minimises risk of bias) across studies to allow effective comparison of the magnitude of adherence. This should distinguish between intentional and unintentional adherence, using measures such as the MARS or MMAS-4, which include questions designed to measure both intentional and unintentional adherence. Consistency in how adherence is defined and measured will greatly facilitate the development of a cumulative evidence base. Such an evidence base is required to help clinicians understand the reasons behind non-adherence and help them build a positive relationship with breast cancer survivors, aiding them to persevere with HT treatment [25].

Future studies should distinguish clearly between adherence and persistence. Persistence measurement should consider the planned duration for the individual patient, which in many cases will be longer than 5 years, to reflect changes to the prescription duration recommendations [89,95]. However, recent qualitative research [96] indicates that taking HT for 10 years is especially difficult. Our recent systematic review [25] highlighted the dilemma breast cancer survivors’ face in balancing the benefits of long-term treatment with the impact of side effects on their quality of life. Therefore, the impact of side effects on long-term persistence with HT must be considered.

Validated self-report measures should be used to capture the range of severity and symptom burden people may experience when taking HT, and consider the impact of individual side effects rather than group them into one homogenous category. This would allow comparison between levels of side effect severity, rather than simply the presence or absence of side effects. A combination of solicited and unsolicited side effect measures should also be considered. Asking patients open-ended questions may encourage them to report a wider range of side effects, as they will not be limited to discussing those suggested by the clinician/researcher. In addition, asking about specific side effects could encourage people to report things that they would not otherwise report [97,98]. A combination of solicited and unsolicited measures would therefore help us to capture the experience of HT side effects more fully, and potentially gain insight into the experience of different HT drugs. Identifying the most troubling side effects would help select appropriate targets for behaviour change interventions. As our recent qualitative review [25] found that BC survivors are highly motivated and willing to incorporate lifestyle changes to manage HT side effects, identifying targets for behaviour change may be an opportunity to facilitate HT adherence in this population.

The side effects of specific HT drugs, and additional treatments such as ovarian suppression, must be considered within future research. Historically, Tamoxifen was the only HT drug suitable for pre-menopausal patients. However, in recent years younger patients have been prescribed a combination of AIs and ovarian suppression. The application of AIs and ovarian suppression in pre-menopausal patients allows potentially greater recurrence prevention than Tamoxifen [99], however, clinicians must balance the benefits of this with the potential side effects [89]. There is a lack of clarity regarding the associations between side effects of different types of HT with adherence and persistence. Future research should therefore evaluate the side effects of specific HT drugs and additional treatments to identify appropriate targets for intervention. Understanding how the profile of side effects that impact on adherence and persistence varies for each class of drug may allow clinicians to inform patients’ expectations more effectively, thus preparing them for the potential consequences and aiding their adherence and persistence.

## Funding sources

This work was supported by a 10.13039/501100000589Chief Scientist Office Catalytic Research Grant (CGA/19/62).

## Declaration of competing interest

The authors have no conflicts of interest to disclose.

## Abbreviations

AI: Aromatase Inhibitor
SERMS: Selective Estrogen Receptor Modulators
HT: Hormone Therapy
