# Supplementary material for: The impact of medication side effects on adherence and persistence to hormone therapy in breast cancer survivors: A quantitative systematic review
Source: Breast. 2022 May 14;64:63–84. doi: 10.1016/j.breast.2022.04.010 (PMC9130570; doi:10.1016/j.breast.2022.04.010)
Supplement: Multimedia component 1 [file mmc1.docx]

# Search Strategy

| Patient Population | Interest | Context |
| --- | --- | --- |
| Breast cancer patients on adjuvant endocrine/ hormonal therapy | Adherence | Impact of side effects |
| breast cancer*  breast neoplasm*  Breast Neoplasm  Breast Carcinoma  Adjuvant breast cancer  Breast cancer survivor  Breast cancer survivorship Survivor  Survivorship  Tamoxifen  Anastrozole  Arimidex  Exemestane  Aromasin  Letrozole  Femara  Hormonal therapy  Aromatase inhibitor* hormon* therapy  Endocrine therapy*  Selective estrogen receptor modulators  SERM  Adjunctive Treatment Adjuvant Treatment | Medication adherence  Patient Compliance  adher*  complian*  persist*  discont*  nonadherence  non-adherence  noncompliance  non-compliance  Non-persistence | Adverse effects  Side effects  Vasomotor  Night sweats  Hot flushes  Musculoskeletal  pain  bone pain  Vulvovaginal  menopausal  gynaecological  sex  weight  weight gain  loss of appetite  social function  Quality of life  cognitive dysfunction  fatigue  Sleep*  sleep problems  insomnia  depression  anxiety  mental wellbeing |

| Database | Dates | Search Terms |
| --- | --- | --- |
| Medline |  |  |
| PsycINFO |  |  |
| Cochrane Library |  |  |
| Embase |  |  |
| Web of Science |  |  |
|  |  |  |
